# 3D Radiation Pattern Reconfigurable Phased Array for Transmission Angle Sensing in 5G Mobile Communication

**DOI:** 10.3390/s18124204

**Published:** 2018-11-30

**Authors:** Jin Zhang, Shuai Zhang, Xianqi Lin, Yong Fan, Gert Frølund Pedersen

**Affiliations:** 1 School of Electronic Science and Engineering, University of Electronic Science Technology of China, Chengdu 611731, China; jzhang@es.aau.dk (J.Z.); yfan@uestc.edu.cn (Y.F.); 2 Department of Electronic System, Aalborg University, 9220 Aalborg, Denmark; gfp@es.aau.dk

**Keywords:** radiation pattern reconfigurable antenna, phased array, 5G mobile communication, transmission angle sensing

## Abstract

This paper proposes a 3D radiation pattern reconfigurable antenna (RPRA) and a reconfigurable phased array (RPA) for 5G mobile communication. The antenna and array are working at 28 GHz, which is selected as a 5G communication band in many countries. The proposed phased array will be applied as sensors to find out the optimal transmitting–receiving angle in a randomly changed cellular wireless scenarios. The RPRA and RPA are fed by Substrate Integrated Waveguide (SIW) and have three switchable radiation modes: Broadside 1, Broadside 2 and Endfire. The three modes correspond to three different radiation patterns and each of them covers a different area in the Azimuth plane. An eight-element phased array constructed by the proposed RPRA, which is able to switch beam in Azimuth plane and scan in the Elevation plane, is also presented in this paper. The proposed RPA is able to provide much higher spatial coverage than the conventional phased arrays and without additional feeding and phase shifting networks. The beam switching is realized by the PIN diodes. The proposed antenna and array have planer structures and require small clearance on the ground plane which makes them compatible with mobile phones. The simulations show good performance for both RPRA and RPA.

## 1. Introduction

The millimeter wave (mm-wave) band has been considered for the upcoming fifth generation (5G) communication for its wideband and high channel capacity properties [1,2]. Under the premise of a larger information capacity, 5G aims to extend the network to a higher level which is composed of both users’ interaction and massive Internet of Things (IoT) [3,4]. The signal propagation mode is investigated by the system models of 5G cellular networks and measurements [1,5,6]. Due to the high path loss of mm-wave, high gain antennas [7] are chosen to compensate for the loss and thus beamsteering arrays are necessary for building an optimal communication link between the user and the base stations. Considering the complicated environment in real life, the real-time connection requires the mobile antennas to have a wide scan angle. This makes spatial coverage one of the most important indexes for mobile phone antenna design. For the 5G mobile antennas, only the array pattern is not enough to describe the coverage performance, so the concept of coverage efficiency and the total scan pattern are introduced to evaluate the spatial coverage performance of an array [8]. The coverage efficiency describes the spatial coverage with respect to a given gain level and the total scan pattern shows which area is covered by all possible beams.

Many works on phased arrays and beam switching arrays are done for 5G mobile applications recently. The phased array with wide scanning angle [9] has the advantage to increase the spatial coverage. The beam switching arrays can be achieved with the butler matrix [10,11], which can be realized by different types of the transmission line. The phased array has been designed with the metallic case of a mobile phone to better satisfy the size limitation [12]. The studies on dual-polarization arrays [13] and decoupling techniques [14] are suitable for Multi-Input Multi-Output (MIMO) systems to further improve the channel efficiency. The tunable Orbital Angular Momentum (OAM) beams array [15] also has potential in 5G mobile communication for its property of decreasing the channel crosstalk.

To further increase the coverage efficiency, multiple subarrays are implemented and each of them are expected to cover a desired area [16,17,18]. However, more subarrays require more feeding ports which increase the loss and system complexity. Nonetheless, the idea of combining beam switching with the phased array is still a realistic method to reach higher spatial coverage. Instead of subarrays, reconfigurable antennas are able to switch beam with only one feeding port. Many works have been done on RPRA. One method is to introduce parasitic elements [19,20]. Switches such as PIN diodes or MEMS are used to connect the parasitic elements with active part. Since only part of the antenna is effective for one mode, the aperture of the whole antenna is not fully used. Because of the low aperture efficiency, the antenna size is always larger compared to the $\lambda_{0}/4$ of working frequency. It may not cause problems in an unlimited space scenario but the space for antennas in mobile phones is very limited. Moreover, to reach higher scan angle, the element distance in a phased array should be no more than $\lambda_{0}/2$. Another method is to introduce reconfigurable reflectors to a fixed radiator [21,22,23]. It is able to have multiple beams and cover a whole plane but it always requires 3D structure and complex controlling system which are very difficult to realize as an array element. As a conclusion, the RPAs for mobile phone applications are expected to have a small size, planar structure, simple feeding and easy control.

In this paper, an RPRA and the phased array constructed by the same reconfigurable array elements are proposed. By combining beam switching in Azimuth plane and beam scanning in the Elevation plane, the spatial coverage is improved without introducing additional feeding ports. The paper is organized as follows: First, the RPRA is introduced from its working principle to the simulation results. Second, the performance of applying this reconfigurable antenna into an eight-element array is presented. Then, it is finished with conclusions.

## 2. Radiation Pattern Reconfigurable Antenna (RPRA)

**Figure 1:**
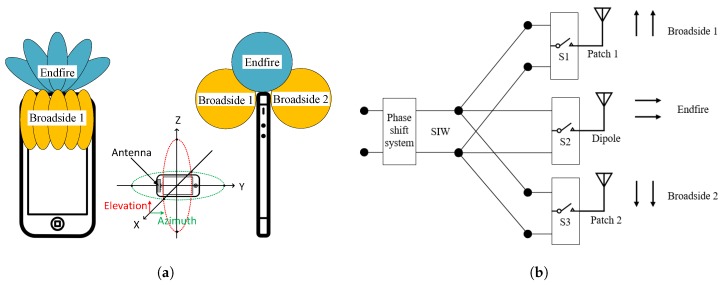
Working principle of the proposed RPRA and RPA: (**a**) the idea of reconfigurable phased array in mobile phone; and (**b**) the schematic of feeding and controlling of the proposed RPRA.

The concept of the reconfigurable phased array is shown in Figure 1a. This array is assumed to be installed on the top of a mobile phone with three switchable modes: Broadside 1, Broadside 2 and Endfire. In each mode, it works independently as a phased array. The design of the RPRA is introduced in this section.

### 2.1. Working Principle

The antenna design is based on SIW for its many advantages in mm-wave antenna design. First, it has a planar structure and is easily fed by both waveguide and coaxial cable. Second, SIW supports many antenna types with either broadside or Endfire radiation patterns. In our design, the RPRA is fed by SIW and constructed with three radiators and three switches, as shown in Figure 1b. The three radiators named as Patch 1, Dipole and Patch 2 correspond to the three radiation modes: Broadside 1, Endfire and Broadside 2. Each mode covers a desired area, as shown in Figure 1a. The switches are built up by PIN diodes and they are controlled by only one DC source for one switch. The RPA is constructed by connecting several RPRAs with one phase shift network, and then the beam scanning of each mode is achieved.

### 2.2. Antenna Structure

**Figure 2:**
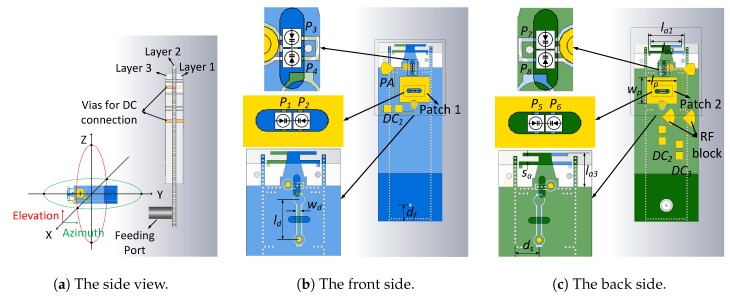
The structure of proposed antenna.

**Figure A1:**
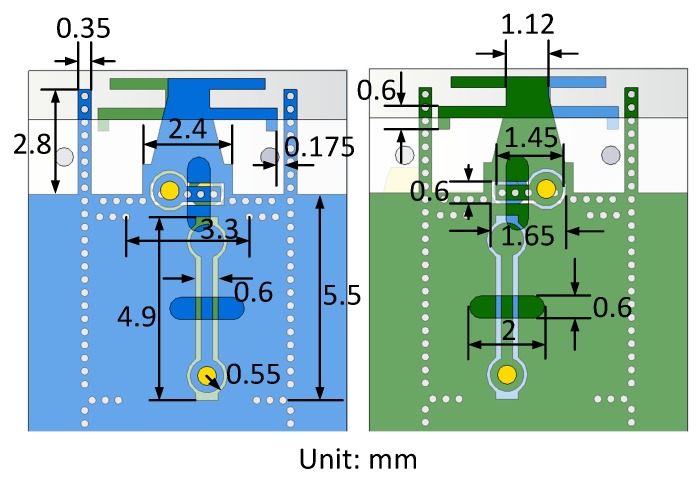
The detailed dimensions of the proposed RPRA.

The antenna is constructed of three layers. The stack-up can be seen from the side view in Figure 2a. The material of the three layers has the same dielectric constant 2.2 and loss angle 0.002. The thickness of Layers 1 and 3 is 1 mm and for Layer 2 is 0.508 mm. The printed structures on the three layers are shown in Figure 2b,c. The surfaces of Layers 1 and 3 are shown as yellow and both sides of Layer 2 are blue and green. The light blue and light green represents the area of Layers 1 and 3, respectively. All the PIN diodes are mounted on Layer 2. Two cylindric holes are made on Layers 1 and 3 above the PIN diodes for fabrication requirements. Patch 1 and Patch 2 are fed by energy coupling through slots. Dipole, which has its two arms printed on both sides of Layer 2, is fed by the SIW directly. The whole antenna is fed by coaxial cable through a probe transition to SIW. Some key dimensions are listed in Table 1. Apart from the DC feeding lines, the whole structure is rotationally symmetric. The other detailed dimensions are listed in Appendix A, Figure A1.

Some design considerations are listed below:

- Patch 1 and Patch 2: The slots on SIW are able to radiate with broadside radiation patterns as well as patches. However, by introducing the patches as second radiators, the bandwidth is improved. Moreover, the impedance matching of the broadside modes is influenced by both the slots and the patches, which gives more freedom for the design. For example, more changes can be done on the patches instead of the slots. Since the slots are etched on the SIW, fewer changes lead to less influence on the other modes. It is an advantage in this RPRA design, which guarantees the design of the three modes independently to a certain degree. On the other hand, the additional patches do not increase the complicity of the whole antenna because multilayer structure is anyway needed for the DC feeding network.
- Dipole: The Dipole is fed by the SIW with a trapezoid shape transition. The arms are composed of two parallel strips for improving the bandwidth. The metallic via walls of the SIW is extended to the Dipole direction to improve isolation between the adjacent Dipoles for the array scenarios.

### 2.3. Beam Switching

The beam switching with PIN diodes is introduced in this section. As mentioned before, there are three switches corresponding to the three modes. Each switch is built by a set of PIN diodes. The low capacitance PIN diode MA4AGBL912 ( the datasheet is available at [24]), which works until 40 GHz, was selected for this antenna design. The parameters for building its equivalent circuits are from the PIN diode datasheet. It shows that, when the PIN diode is on, it equals to the series resistance of $R_{s}=4.2$ Ω and inductance of $L_{s}=0.5\;\mathrm{nH}$, while, when the PIN diode is off, it equals to the series capacitance of $C_{t}=28\;\mathrm{fF}$ and inductance of $L_{s}=0.5\;\mathrm{nH}$.

In Figure 1b, the PIN diodes are represented by switches $S_{1}$, $S_{2}$, and $S_{3}$. All the PIN diodes are mounted on both sides of Layer 2. They can be seen from both front and back side through the holes as shown in Figure 2b,c. The correspondence of the switches and the PIN diodes are: $S_{1}$ corresponds to $P_{1}$ and $P_{2}$; $S_{2}$ relates to $P_{3}$, $P_{4}$, $P_{7}$, and $P_{8}$; and $S_{3}$ is $P_{5}$ and $P_{6}$. A dumbbell shape strip is inserted in the middle of the slot for the DC supply which connects to the dumbbell strip through a metallic via. In Figure 2b,c, the yellow dots represent the connection points. The positive ends of $P_{1}$ and $P_{2}$ are connected to the dumbbell strip and the negative ends solder on the SIW. Then, $P_{1}$ and $P_{2}$ have the same on/off state with the control of $DC_{1}$. This is the same for $P_{5}$, $P_{6}$ and $DC_{2}$. Since the patches are fed by the slots through energy coupling, the control of slots equals the control of patches. For instance, when $P_{1}$ and $P_{2}$ are on, the slot is not resonant and little power is coupling to Patch 1. In this case, Patch 1 will not radiate. On the contrary, when the PIN diodes are off, the slot is resonant and then Patch 1 will radiate. The positive ends of the four PIN diodes of $S_{2}$ are connected to an isolated small wall of metallic vias, which connects $DC_{3}$ through a via. When they are on, the via wall is connected with the SIW, so the energy is stopped inside the SIW and the dipole antenna does not radiate. On the other hand, when they are off, the via wall is isolated with SIW; the energy will pass it and radiate by the Dipole. The status “on” and “off” means opposite for switches and PIN diodes here. For example, as in Figure 1b, when $S_{1}$ is “on” while $S_{2}$ and $S_{3}$ are “off”, only Patch 1 or Broadside 1 mode is working. In this case, the PIN diodes status in Figure 2 is that $P_{1}$ and $P_{2}$ are off while the others are on. To ease of understanding, the switch status and PIN diodes status are summarized in Table 2. Another thing to be mentioned is the parasitic structure PA, as shown in Figure 2b. Its shape is the same as part of $DC_{3}$ and it is rotationally symmetric with $DC_{3}$. Both PA and $DC_{3}$ are connected to the via wall but PA is not connected with any DC supply. It acts as a passive loading of the Dipole which balances the currents on both arms. Consequently, the radiation pattern of the Dipole becomes more symmetric.

### 2.4. The Simulation and Analysis

This section includes a design procedure, parameter analysis and the simulation results of the proposed RPRA. All simulations in this paper are done in CST STUDIO SUITE 2018.

#### 2.4.1. Design Procedure

A brief design procedure is provided below. It can also be used as a guideline for others who want to design a similar structure for their own purpose.

- Step 1: Decide the SIW size according to the working frequency. The width of SIW should allow the lowest frequency to propagate and suppress the high order modes of the highest frequency.
- Step 2: Design the slots and dipole antennas separately at the working frequency band. For the slots, an inductive window is introduced to form a resonant cavity. The opening width of the window controls the energy coupling from the transmission line to the cavity.
- Step 3: Combine the slots and dipole within one SIW. The PIN diode effects should be considered in this step because the different loads lead to different input impedances. A compromise of impedance matching has to be made for all three modes.
- Step 4: Add the layer with patches. Increasing the thickness of the patch layers also increases the bandwidth of the broadside modes. The additional material has slight influence on the dipole. Adjust the size and position of the patches to get the best impedance matching for the broadside modes.
- Step 6: Add DC feeding lines and vias. Adjust the size and position of the sector patches to get the best RF blocking.

**Figure 3:**
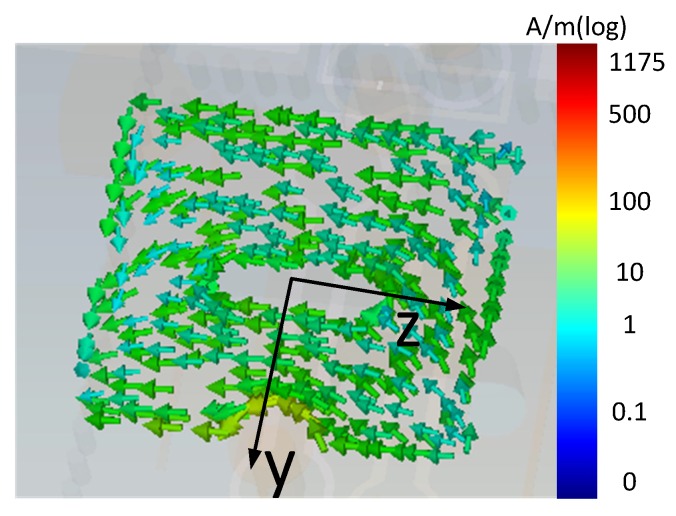
The surface current distribution on the working patch.

In our design, the SIW is fed by a coaxial cable with a probe transition. The width of the SIW is chosen as 5.5 mm according to the half-wavelength of 28 GHz. The diameter and distance of the vias for SIW are 0.2 mm and 0.35 mm, respectively. The length of the resonant cavity is 5 mm. The opening width of the inductive window is 3.5 mm. The cylindrical holes on Layers 1 and 3 are made for not smashing the PIN diodes when the three layers are stacked. The surface current direction is along *z*-axis at 28 GHz, as shown in Figure 3. The holes in the middle of the patches are oriented along with the current direction, which can help to reduce the current disturbing on the patches. The subsequent simulations show that the appearance of the holes brings slightly size reduction of the patches.

#### 2.4.2. Parameter Analysis

**Figure 4:**
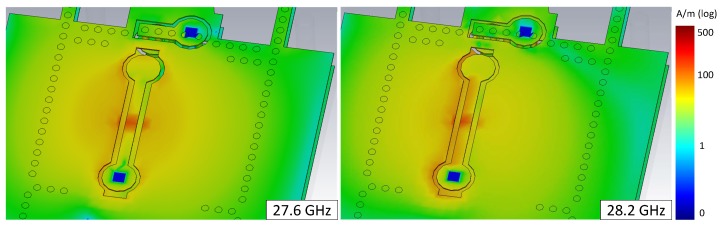
The current distribution on SIW of the two resonances.

**Figure 5:**
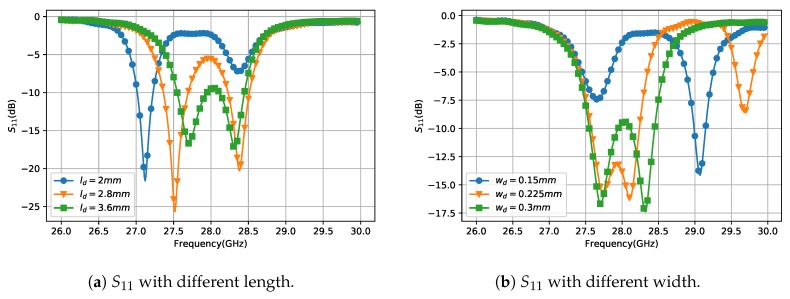
The influence of the dumbbell shape strip on $S_{11}$.

The SIW cavity-backed slot antenna was first proposed by Luo [25]. The cavity resonants at $TE_{120}$ mode and the slot radiates by cutting the transverse electric field. In our design, a dumbbell shape strip is inserted in the middle of the slot for soldering the PIN diodes and connecting the DC feeding line. The appearance of this strip turns the slot into a loop, which, as a consequence, supports two different resonances at 27.6 GHz and 28.2 GHz. The surface current distributions are shown in Figure 4. The current at 27.6 GHz distributes on both side of the loop but at 28.2 GHz the current is only on the left side. It means that at 27.6 GHz the whole loop takes part in the resonance while at 28.2 GHz only the slot between dumbbell strip and the SIW takes part in the resonance. Therefore, the two resonances can be controlled by only changing the dumbbell strip. The influence of the dumbbell strip on the resonance frequencies is shown in Figure 5. In Figure 5a, the width of the strip $w_{d}=0.3\;\mathrm{mm}$ and the length $l_{d}$ changes from 2 mm to 3.6 mm. In this case, the resonance at higher frequency does not move but the frequency of the lower resonance increases as $l_{d}$ increasing. In Figure 5b, the length of the strip $l_{d}=3.6\;\mathrm{mm}$ and the width $w_{d}$ changes from 0.15 mm to 0.3 mm. As the strip becomes wider, the slot width on the loop becomes narrower. In this case, the lower resonance does not move. The frequency of higher resonance decreases when $w_{d}$ increases from 0.15 mm to 0.225 mm but increases again if $w_{d}$ keeps increasing from 0.225 mm to 0.3 mm. Therefore, by tuning the shape of the dumbbell strip, the two resonances can be controlled separately to find the best balance of impedance matching and the bandwidth.

#### 2.4.3. Results

The influences of the connector and DC feeding lines are considered in the final simulation results. The DC feeding lines are shown in Figure 2. The RF blocks have to be added on the DC feeding lines to suppress the RF signal leaking which can cause impedance mismatching and generate unwanted radiation. Without the RF blocks, the radiation of the DC feeding lines could be significant at 28 GHz. As a result, it will lead to radiation patterns distortion and increase the loss.

**Figure 6:**
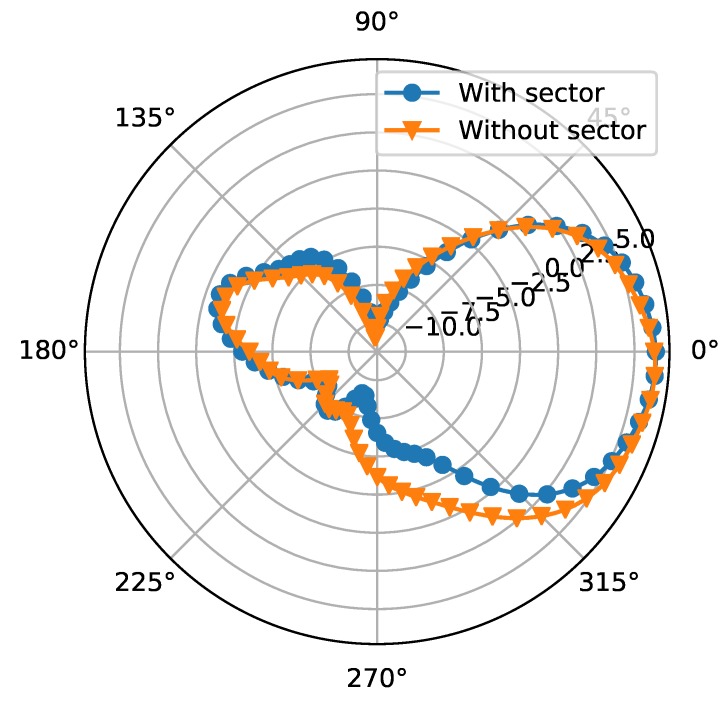
The radiation pattern of Patch 1 with or without the RF block sector on $DC_{1}$.

**Figure 7:**
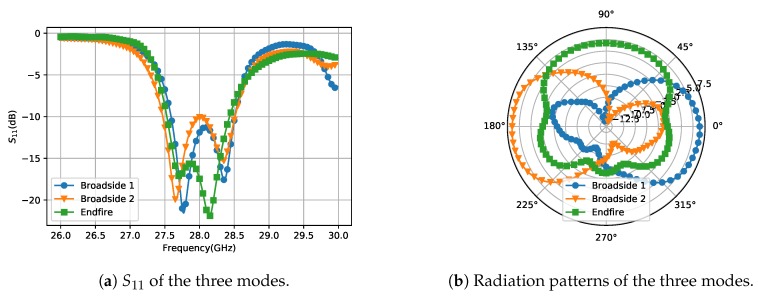
The $S_{11}$ and radiation patterns of the three modes.

Therefore, some sector patches are introduced as RF blocks. The radius of the sectors is λ/4 of 28 GHz to make an equivalent short point at center frequency. The distance from the sector patch to the DC feeding point should also be λ/4+Nπ so that the feeding point will be equal to open. To leave more space for the screen and camera, the antenna size, including the DC feeding lines, is expected to be as small as possible on at least one side of the ground plane. Therefore, $DC_{2}$ and $DC_{3}$ are designed longer and two sector patches on $DC_{3}$ are adopted to improve the blocking effect. On the other hand, to reduce the occupied space, $DC_{1}$ is designed very short and no RF block is applied. In this case, the RF signal on $DC_{1}$ will be partially consumed by the DC current limiting resistor and partially be radiated. To investigate how much power is lost on $DC_{1}$ without RF block, the simulated radiation pattern of Patch 1 with and without the sector is given in Figure 6. The realized gain of the two cases are not significantly different which means the energy consumed by the resistor is very low. The radiation of $DC_{1}$ is reflected in the shape of the radiation pattern. The one without sector shows higher gain from $270^{\circ }$ to $315^{\circ }$. The absence of RF block influence not so much, which is mainly because the the capacitance between the square patch and the ground is enough to block the RF signal in this case. Even though the loss on the DC feeding lines is reduced by adding the RF blocks, the impedance matching can still change a little because of the inductance of DC feeding lines. However, it can be compensated easily by adjusting the position of SIW feeding port. The reflection coefficiency and H-plane radiation patterns at 28 GHz of the three working modes are shown in Figure 7. Two resonances are observed in all three modes, as shown in Figure 7a. The overlapped 6-dB bandwidth is 3.9% from 27.5 GHz to 28.6 GHz. Figure 7b shows the H-plane radiation patterns of the three modes on the Azimuth plane. The overlapped 3-dB beamwidth is up to $270^{\circ }$. The realized gain for Broadside 1, Broadside 2 and Endfire mode are 6.5 dBi, 6.6 dBi, and 4.3 dBi, respectively.

## 3. Design of Eight-Element Array

**Figure 8:**
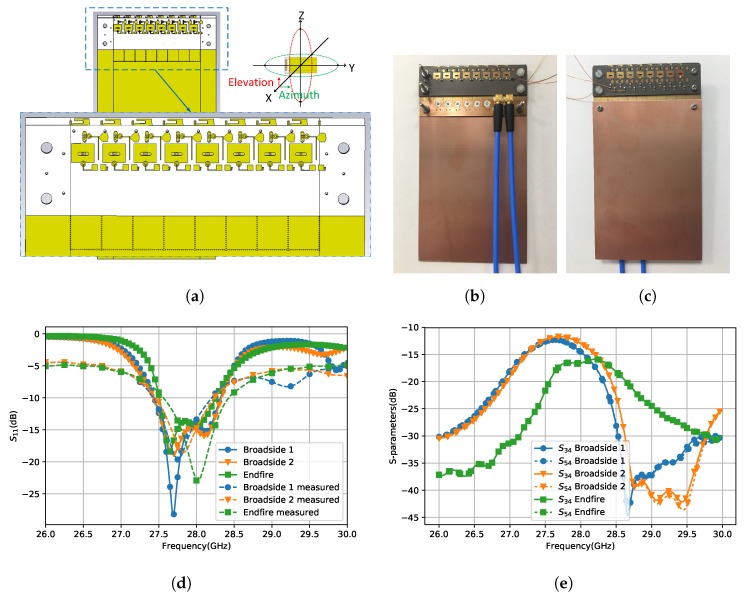
The array structure and the experiment results of the middle element: (**a**) the array structure of the front view; (**b**) front view; (**c**) back view; (**d**) the simulated and measured reflection coefficiency of the middle element; and (**e**) the simulated mutual coupling of the middle element.

**Figure 9:**
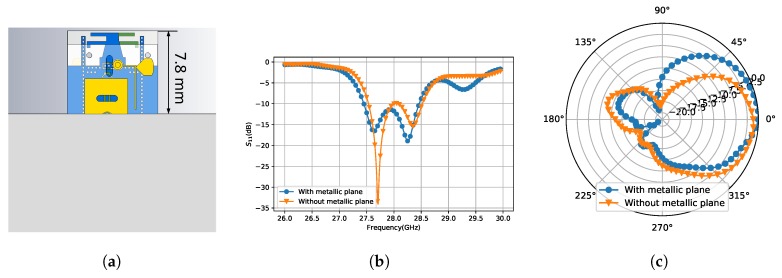
The influence of adjacent metallic plane: (**a**) the array element with a metallic plane above; (**b**) the $S_{11}$ of Broadside 1 with or without the metallic plane; and (**c**) the radiation pattern of Broadside 1 with or without the metallic plane.

In the last section, the radiation pattern reconfigurable antenna is proved to be able to provide a wide coverage on the Azimuth plane. For the Elevation plane, the coverage is improved by beam scanning. The number of the array elements was chosen as eight in this design. The array was simulated on a 60mm×110mm ground plane which represents the ground plane of a mobile phone as shown in Figure 8a. The antenna was fabricated and measured on the ground plane of the same size. The front and back view of the fabricated model is shown in Figure 8b,c. The distance between array elements is 5.5 mm, which is the same as the SIW width and λ/2 of 28 GHz. The clearance which the array needs on the ground plane is 9.25mm×44mm. It means that the antenna performance will not be affected by other components if the antennas are within this area. A metal plane was added 0.5 mm above the antenna element to investigate the influence of the adjacent metallic objects, as shown in Figure 9a. This metal plane covers the DC feeding line and the edge of Patch 1. The space left on the top is 7.8 mm, which is smaller than the top clearance of most of the current mobile phones. The simulated $S_{11}$ and radiation patterns with and without the metal plane are in Figure 9b,c. The antenna with the metal plane still shows good impedance matching in the working band. It has more radiation on the Endfire direction but the main beam direction remains on the broadside. In the paper, the clearance of the proposed antenna is stated as 9.25 mm because we count also the space of DC feeding line but it can be covered by other metal objects without bringing too much influence to the antenna performance.

Figure 8d shows the simulated and measured reflection coefficiency of the three radiating modes of the fourth element which is in the middle of the whole array. The simulation and the measurement have good agreements. The overlapped 10-dB bandwidth is 2.68% from 27.5 GHz to 28.3 GHz which is sufficient to cover the band of one channel. In industry, 6-dB bandwidth can also be accepted. The overlapped 6-dB bandwidth is 6.6% from 27 GHz to 28.9 GHz (over 1 GHz). The results of other array elements are not shown here for simplicity because all of them have similar performance. The highest mutual coupling happens between the adjacent elements. The mutual coupling of the middel element and its adjacent elements are shown in Figure 8e. It is below −11 dB for the broadside modes and below −15 dB for the Endfire mode. The coupling of the Endfire mode is lower because the extending via walls between the dipole elements improve the isolation. The increment of mutual coupling will decrease the scanning angle and as a consequence decrease the spatial coverage.

**Figure 10:**
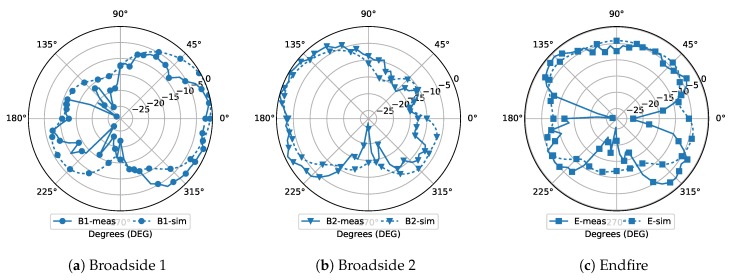
The simulated and measured radiation patterns with copper wire.

The array is measured with the PIN diodes for the S-parameters. However, some PIN diodes were broken during the measurement and we failed to put them back because they are too small and fragile to operate by hand. Unfortunately, we do not have a professional machine to mount very small components. Thus, to find a way for verifying the radiation patterns, we used copper wires to replace all the PIN diodes. We measured the radiation patterns at 29 GHz instead of 28 GHz because the antennas are mismatched when the PIN diodes are replaced by copper wires. The antenna design depends on the PIN diode’s equivalent circuits to compensate its parasitics’ effects. When the copper wires were used, the parasitics change dramatically from the simulation model, which led to a big change on impedance matching and shifting of resonant frequencies. However, the measurement of copper wire can still give a view of the reconfigurable radiation patterns. Figure 10 shows the measured radiation patterns of the three modes with the copper wires. The two broadside modes (Figure 10b,d) have the main beam direction at $10^{\circ }$ and $165^{\circ }$ in the measurement. The directivity of the Endfire mode (Figure 10c) drops at $90^{\circ }$, so its main beam is at $135^{\circ }$ in the measurement. This is mainly because the variation of the parasitics also influences the radiation pattern. Ideally, the impedance of the working mode is completely matched with the input impedance while the non-working modes are completely mismatched, so the energy will only radiate through the working mode. However, when the working mode is also mismatched, the impedance of the working mode and the non-working modes may get closer. Considering that a big amount of energy is reflected, the leaking energy through the non-working modes is growing comparing with the radiation through the working mode. Therefore, the Endfire radiation pattern has less directivity at the $90^{\circ }$ and has more radiation at $45^{\circ }$ and $135^{\circ }$. The Endfire radiation pattern is a little distorted but it is more important that the simulation and the measurement results in Figure 10c are matching very well. It proves that the simulation models are built very close to the real scenario. If the structure were made correctly with PIN diodes, the measured radiation patterns are expected to be similar to the simulation. In practical applications, this kind of structure can be made as a whole in the industry, where the multi-layer PCB and PIN diode mounting will be done at one time.

**Figure 11:**
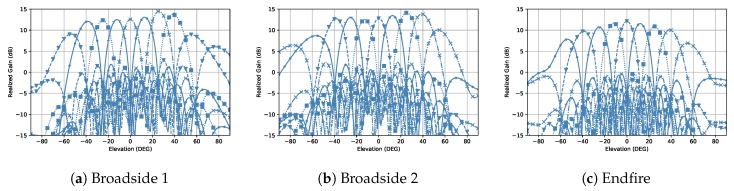
The array scan pattern on the Elevation plane.

Phase shifters can be used for beam scanning. For instance, previous studies refs. [26,27] show 4-bit and 5-bit phase shifters at 28 GHz with CMOS technology, which provide up to $360^{\circ }$ phase difference with low insertion loss and very compact size. Figure 11 gives the array scan pattern on the Elevation plane of the proposed three modes. For each mode, nine beams are sufficient to cover the scanning range, which is from $-53^{\circ }$ to $55^{\circ }$ for Broadside 1 (Figure 11a), from $-56^{\circ }$ to $54^{\circ }$ for Broadside 2 (Figure 11b), and from $-54^{\circ }$ to $54^{\circ }$ for Endfire (Figure 11c), respectively.

**Figure 12:**
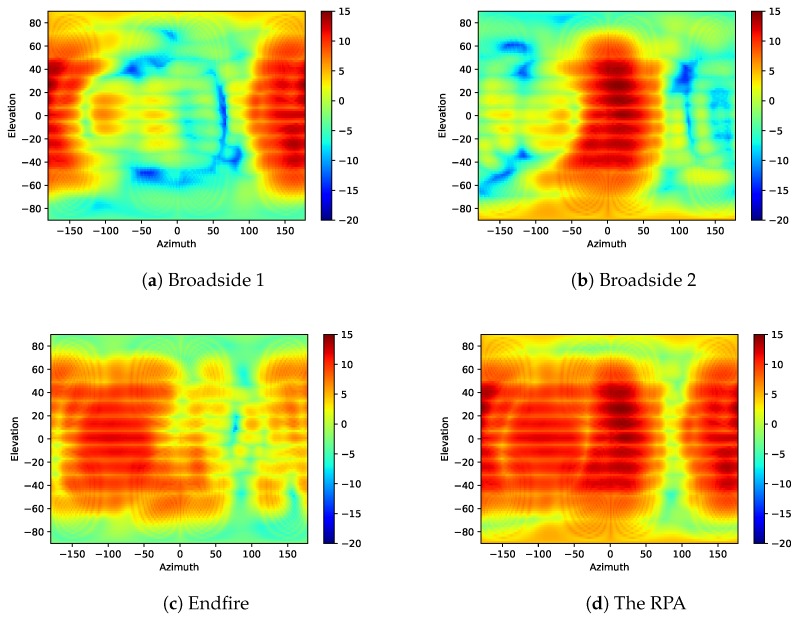
The total scan patterns of every single mode and the RPA.

The total scan pattern was studied by the simulation results, which Was used to evaluate the gain and spatial coverage performance of a phased array. It was obtained by extracting the highest gain at every point of all steered beams [8]. Figure 12 shows the total scan pattern of every single working mode and the three modes together. The two broadside modes, as shown in Figure 12a,b, have similar scan patterns.The main beam located at $180^{\circ }$ and $0^{\circ }$ in the Azimuth plane, respectively. The Endfire mode, as shown in Figure 12c, has the main beam at $-90^{\circ }$. In the Azimuth plane, the Endfire mode represents higher beamwidth, higher backward radiation and lower peak gain. The total scan pattern of all three modes are shown in Figure 12d with 27 steered beams which gives a intuitive understanding of how much the spatial coverage is improved by the proposed RPA. It is obvious that the RPA has much higher and more uniform coverage than any single mode. As we can see in Figure 12d, a null appears at $90^{\circ }$ in the Azimuth plane, which corresponds to $270^{\circ }$ in Figure 7b. If we see the array in the coordinate system in Figure 8a, the null appears at +*y*-direction, which is the opposite of the Endfire direction. Because of the ground plane, this direction is difficult to cover with the radiation patterns of the proposed array.

**Figure 13:**
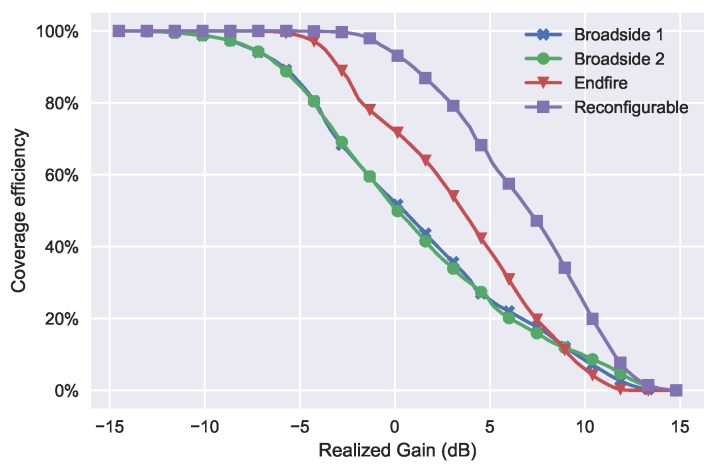
Coverage efficiency of the RPA and every single mode.

The coverage efficiency is calculated by the coverage solid angle and the maximum solid angle [8]. When a certain threshold of gain is given, it comes out of a percentage of the covered area with gain above the threshold from the whole plane. Compared with the total scan pattern, the coverage efficiency is a quantitative indicator of the spatial coverage. Figure 13 shows the coverage efficiency of the proposed RPA and the three single modes as a comparison. The result was calculated by the simulated array patterns with PIN diodes. As we can see, the two broadside modes have the same coverage efficiency, while the Endfire mode shows higher coverage below 8 dBi gain. The reconfigurable curve represents the total coverage efficiency with all three modes. The figure also shows us how much spatial coverage can be improved with RPA. The curves of every single mode can be seen as one conventional phased array with either broadside or Endfire radiation pattern. Compare between the single modes, if only one phased array is used in mobile phone, then Endfire arrays will provide higher coverage than broadside arrays. However, the spatial coverage is significantly improved with radiation pattern reconfigurable techniques. The coverage efficiency at 0 dBi is more than 90% for the RPA, while it is around 70% for Endfire array and 50% for broadside array. The coverage efficiency at 5 dBi is around 65% for the reconfigurable array, 40% for the Endfire array and only 25% for the broadside array.

## 4. Conclusions

This paper proposes an RPRA with three switchable radiation patterns which cover three different areas in Azimuth plane. The 3D spatial coverage is achieved by using the proposed RPRA as an array element for an eight-element phased array. Both RPRA and RPA are working at 28 GHz for the 5G communication. The proposed antenna and array are suitable for use in the sensors for mobile terminals to automatically detect and connect with the base stations. Because of the planar structure and small clearance, they are compatible with the thinner thickness and the bigger screen of the mobile phones. The simulated and measured results verified our theory. The antenna and array were fabricated with multi-layer PCB and the PIN diodes were embedded in the PCBs. Even though we faced many problems dealing with the PIN diodes in our lab, it is not a problem in industry. Since both techniques are mature and widely used nowadays, the complexity and cost of the mass production will not be so high. Moreover, the feeding and DC control networks can be integrated with the subsequent circuits, which may save more space than our proposed design.

## Figures and Tables

**Table 1:** The key dimensions in the antenna model (unit: mm).

$d_{f}$	$l_{d}$	$w_{d}$	$d_{s}$	$s_{a}$
1.5	1.8	0.3	2.23	0.5
${\mathrm{la}}_{1}$	${\mathrm{la}}_{2}$	${\mathrm{la}}_{3}$	$w_{p}$	$l_{p}$
4.8	4.14	3.1	3.4	4

**Table 2:** The switch and PIN diodes status for different working modes.

Working Modes	$S_{1}$	$P_{1}$, $P_{2}$	$S_{2}$	$P_{3}$, $P_{4}$, $P_{7}$, $P_{8}$	$S_{3}$	$P_{5}$, $P_{6}$
Broadside 1	on	off	off	on	off	on
Endfire	off	on	on	off	off	on
Broadside 2	off	on	off	on	on	off

## Abbreviations

The following abbreviations are used in this manuscript:

RPRA	radiation pattern reconfigurable antenna
RPA	reconfigurable phased array
SIW	Substrate Integrated Waveguide
mm-wave	millimeter wave
5G	fifth generation
MIMO	Multi-input Multi-output
IoT	Internet of Things

## Appendix A

The detailed dimensions of the proposed RPRA is shown in Figure A1.
